# Declining Kidney Function Is Associated with Altered Erythrocyte Membrane Fatty Acid Profiles and Estimated Enzymatic Indices

**DOI:** 10.3390/membranes16100342

**Published:** 2026-10-14

**Authors:** Jin Kyeong Kim, Dong Eun Yang, Seong Eun Kim, Won Suk An, Su Mi Lee

**Affiliations:** 1 Department of Internal Medicine, Dong-A University, Busan 49201, Republic of Korea; jin0129k@naver.com (J.K.K.); eastin@dau.ac.kr (D.E.Y.); sekim@dau.ac.kr (S.E.K.); 2 Medical Science Research Center, Dong-A University, Busan 49201, Republic of Korea

**Keywords:** chronic kidney disease, oleic acid, linoleic acid, omega-3, fatty acid metabolism, ELOVL6, desaturase

## Abstract

Patients with chronic kidney disease (CKD) face a substantial cardiovascular burden, with altered erythrocyte membrane fatty acids (FAs) representing potential markers of adverse outcomes. However, comprehensive evaluations according to varying levels of kidney function remain limited. This retrospective pooled analysis evaluated the association between kidney function decline, erythrocyte FA profiles, and estimated enzymatic indices (surrogate product-to-precursor ratios) across eight distinct cohorts (*N* = 282: 33 healthy controls, 131 non-dialysis CKD, 118 end-stage kidney disease). Generalized linear mixed models accounted for inter-cohort heterogeneity with study source as a random intercept, adjusting for age, sex, diabetes, and serum albumin. With declining kidney function, erythrocyte membrane oleic acid exhibited a stepwise increase alongside an elevated elongation of very long-chain fatty acids protein 6 index, whereas linoleic acid and the stearoyl-CoA desaturase 18 index progressively decreased. Multivariable models confirmed that estimated glomerular filtration rate was an independent predictor of oleic (β = −0.012, 95% CI: −0.024 to −0.0003, *p* = 0.044) and linoleic (β = 0.016, 95% CI: 0.004 to 0.028, *p* = 0.012) acid levels. In conclusion, kidney dysfunction is independently associated with an altered membrane FA composition, characterized by an increase in monounsaturated FAs and a decline in essential polyunsaturated FAs. These findings suggest a potential link between declining kidney function and altered lipid metabolism, providing a basis for further studies exploring targeted nutritional interventions in CKD.

## 1. Introduction

Chronic kidney disease (CKD) represents an escalating global public health burden affecting over 10% of the general population worldwide [1]. Patients with CKD have an increased risk of cardiovascular disease (CVD), the leading cause of morbidity and mortality in this population [2]. As traditional risk factors fail to account for this excess risk, research attention has shifted toward non-traditional pathways, specifically kidney-specific metabolic disorders. Plasma fatty acids (FAs) reflect dietary intake over only a few days. In contrast, erythrocyte membrane FAs provide a stable long-term marker of habitual status over the 3-month red blood cell lifespan [3,4]. Specifically, the accumulation of oleic acid and a deficit in omega-3 FAs are linked to adverse cardiovascular outcomes [5,6,7]. Progressive kidney damage involves complex pathological cascades characterized by oxidative stress, chronic inflammation, and systemic lipid dysregulation [8,9]. Although CKD alters lipid metabolism, promotes chronic inflammation, and increases oxidative stress [10,11,12], alterations in erythrocyte membrane FA profiles across the continuum of kidney dysfunction have not been systematically evaluated using an integrated multi-cohort design.

FA metabolism is regulated by several enzymatic pathways involving desaturases and elongases. Stearoyl-CoA desaturase (SCD) converts saturated FAs (SFAs) into monounsaturated FAs (MUFAs) such as oleic acid, while delta-6 desaturase (Δ6-desaturase), delta-5 desaturase (Δ5-desaturase), elongation of very long-chain fatty acids protein 5 (ELOVL5), and ELOVL6 mediate the synthesis of polyunsaturated FAs (PUFAs) and regulate carbon chain length [13,14]. In CKD, uremic status disrupts these key enzymatic pathways, altering overall erythrocyte membrane FA composition. Omega-3 FA supplementation can modify this membrane structure and reduce oleic acid levels through membrane FA remodeling [11]. However, specific coordination between the desaturase and elongase pathways during progressive kidney decline has not yet been established.

Prior investigations have evaluated erythrocyte membrane FA compositions in general cardiovascular cohorts or specific kidney disease populations; however, comprehensive comparisons encompassing the entire spectrum, from healthy controls to end-stage kidney disease (ESKD), remain limited [15]. Understanding the adaptation of FA composition and estimated metabolic indices along this continuum is crucial for developing targeted nutritional and metabolic interventions. The present study investigated the differences in erythrocyte membrane FA composition and estimated metabolic indices among healthy controls, patients with CKD, and patients with ESKD using a retrospective pooled analysis integrating eight distinct cohorts.

## 2. Materials and Methods

### 2.1. Data Sources and Study Design

This study is a retrospective pooled analysis of individual participant data obtained from the Dong-A University Medical Center between 2009 and 2019. To ensure a comprehensive comparison across kidney function stages, individual participant data were extracted and pooled from eight distinct clinical study cohorts conducted at the same institution. These included seven previously published studies [16,17,18,19,20,21,22] and one unpublished clinical dataset (approved by the Institutional Review Board, DAUHIRB-18-237). To avoid double-counting and selection bias, overlapping patients were excluded prior to pooling. Detailed descriptions of each source cohort are summarized in Supplementary Table S1. The healthy control group consisted of 33 individuals from a single source cohort, whereas patients with CKD and ESKD were derived from multiple cohorts. For intervention cohorts, only pre-intervention baseline data obtained prior to any study treatment were extracted and included in the analysis.

The primary objective was to determine differences in erythrocyte membrane FA profiles according to kidney function, emphasizing oleic acid levels and the omega-3 index. Changes in desaturase and elongase indices were estimated to identify metabolic imbalances associated with variable estimated glomerular filtration rates (eGFR). Additionally, the relationship between kidney function indicators and FA composition was evaluated. The study protocol was approved by the Dong-A University Hospital Institutional Review Board (DAUHIRB-26-022). The requirement for informed consent was waived because of the retrospective nature of the study. The overall study selection process, inclusion and exclusion criteria, and patient allocation are summarized in Supplementary Figure S1.

### 2.2. Eligibility Criteria

Participants were selected based on the criteria established in the primary studies. The healthy control group comprised adults aged >18 years with an eGFR ≥ 60 mL/min/1.73 m^2^ without proteinuria or history of hypertension, diabetes mellitus (DM), or chronic medication use. The CKD and ESKD groups comprised patients categorized according to kidney function and dialysis status, as defined in the source studies, including those undergoing either maintenance hemodialysis or peritoneal dialysis for at least 3 months. Patients with acute kidney injury, active malignancy, or a history of recent infection or omega-3 supplementation within the previous 3 months were excluded from primary studies.

### 2.3. Laboratory Measurement

Demographic and biochemical parameters were extracted from the electronic medical records. Laboratory data included blood urea nitrogen (BUN), serum creatinine, eGFR, albumin, calcium, phosphorus, hemoglobin A1c, total cholesterol, triglycerides, low-density lipoprotein (LDL)-cholesterol, and high-density lipoprotein (HDL)-cholesterol. All biochemical parameters were processed and analyzed using a routine automated analyzer at the central laboratory of Dong-A University Hospital. Parameters were evaluated from blood samples collected under fasting conditions at a single baseline time point upon enrollment. For patients on hemodialysis, blood samples were obtained immediately prior to the dialysis session, whereas for patients on peritoneal dialysis, samples were collected during routine clinic visits.

### 2.4. Erythrocyte Membrane FA Profiling

The erythrocyte membrane FA content was measured using gas chromatography following previously reported standardized methods [16]. Briefly, erythrocyte samples were methylated with boron trifluoride methanol–benzene at 100 °C for 10 min. FA methyl esters were separated using a gas chromatograph (Shimadzu 2010AF; Shimadzu Scientific Instrument, Kyoto, Japan) fitted with a 100 m SP2560 capillary column (Supelco, Bellefonte, PA, USA) and quantified against commercial standard mixtures (GLC-727; Nu-Chek Prep, Elysian, MN, USA). All laboratory analyses across the pooled datasets were conducted at a single central laboratory using identical equipment, reagents, calibration protocols, and quality control procedures. To mitigate interstudy variability and ensure consistency across datasets, all FA levels were standardized and expressed as a weight percentage (wt%) of total identified FAs. The omega-3 index was defined as the sum of eicosapentaenoic acid (EPA) and docosahexaenoic acid (DHA) in erythrocyte membranes. Endogenous desaturase and elongase indices were estimated using the product-to-precursor ratios as surrogate metabolic indices.

• SCD16 = C16:1n7/C16:0
• SCD18 = C18:1n9/C18:0
• Δ6-desaturase = C18:3n6/C18:2n6
• Δ5-desaturase = C20:4n6/C20:3n6
• ELOVL5 = C20:3n6/C18:3n6
• ELOVL6 = C18:0/C16:0

### 2.5. Statistical Analysis

The eight cohorts were pooled based on consistent clinical definitions for CKD stages and standardized measurement protocols for FA variables. Generalized linear mixed models (GLMMs) were used for primary comparisons to determine differences in erythrocyte membrane FA composition and estimated metabolic indices across clinical groups. To account for latent heterogeneity across study periods and cohorts, the original study source was included as a random intercept, whereas clinical groups and covariates (age, sex, DM, and serum albumin) were treated as fixed effects. Additionally, sensitivity models specifying study source as a categorical fixed effect were evaluated to assess potential structural confounding between source cohorts and clinical stages. The estimated marginal (EM) means derived from the GLMM framework were reported to provide adjusted FA profiles. Furthermore, multivariable GLMMs were performed to identify independent associations between eGFR (evaluated as a continuous variable) and FA levels after adjusting for the aforementioned confounders. To evaluate potential inter-cohort dissimilarity and ensure no single cohort disproportionately influenced the outcomes, a leave-one-cohort-out sensitivity analysis was conducted by sequentially excluding each individual study group. Additionally, potential effect modification by dialysis status (non-dialysis CKD vs. ESKD) was evaluated in the multivariable models, and a subgroup analysis restricted to non-dialysis participants was conducted. Finally, model fit and the proportion of variance explained by the multivariable GLMMs were evaluated using marginal R^2^ and conditional R^2^ according to Nakagawa and Schielzeth. For all fitted GLMMs, a Gaussian distribution with an identity link function was specified, appropriate for continuous FA weight percentages (wt%). Principal regression coefficients (β) are reported with their corresponding 95% confidence intervals (CIs) and *p* values. Changes in FA weight percentages are presented as percentage-point changes to distinguish absolute shift from relative percentage change.

Data are presented as adjusted means ± standard errors (SE) from GLMM or as raw means ± standard deviations (SD) and frequencies (*n*, %), as appropriate. Normality was assessed using the Shapiro–Wilk test, and supplementary group differences were compared using one-way analysis of variance or the Kruskal–Wallis test, as required. All analyses were conducted using SPSS software (version 27.0; IBM Corp., Armonk, NY, USA). Statistical significance was set at *p* < 0.05.

## 3. Results

### 3.1. Baseline Characteristics

Baseline characteristics of the study population are summarized in Table 1. Data from 282 participants were analyzed, comprising 33 healthy controls, 131 patients with non-dialysis CKD, and 118 patients with ESKD. The mean age was significantly higher in the CKD and ESKD groups than in the control group, whereas sex distribution was comparable across all three groups, with males representing 51.8% (*n* = 146) of the total population. The prevalence of DM was significantly higher in the CKD and ESKD groups than in healthy controls. Kidney function markers, including eGFR, declined progressively from controls to CKD and ESKD, with reciprocal increases in serum creatinine and BUN levels. In parallel with advancing kidney impairment, serum phosphorus and uric acid levels increased, whereas hemoglobin and albumin levels decreased significantly across the groups. HbA1c levels also increased progressively, being highest in the ESKD group. Regarding lipid profiles, total, LDL-, and HDL-cholesterol levels were lowest in the ESKD group, while triglyceride levels showed no significant differences among the groups.

### 3.2. Differences in Erythrocyte Membrane Fatty Acid Composition

Erythrocyte membrane FA composition differed significantly according to kidney function (Table 2). Total SFA levels increased stepwise from controls to the CKD and ESKD groups. In contrast, marked alterations in total MUFAs and PUFAs were predominantly evident in patients with ESKD, who exhibited significantly higher MUFA levels and lower PUFA, omega-6, and omega-3 FA levels compared with both healthy controls and non-dialysis CKD patients.

Among individual FAs, erythrocyte membrane oleic acid levels increased progressively with worsening kidney function, whereas linoleic acid levels declined concomitantly. Among omega-3 FAs, alpha-linolenic acid (ALA) levels were lower in both the CKD and ESKD groups than in controls, whereas EPA and DHA levels were lower in the ESKD group. The omega-3 index was lowest, and the omega-6/omega-3 ratio was highest in the ESKD group. The arachidonic acid (AA)/EPA ratio was elevated in both the CKD and ESKD groups relative to healthy controls.

### 3.3. Estimated Fatty Acid Metabolic Indices

Estimated FA metabolic indices, calculated as surrogate product-to-precursor ratios, varied significantly according to kidney function (Table 2). For desaturase indices, estimated SCD16 and Δ6-desaturase indices were significantly lower in both the CKD and ESKD groups compared with healthy controls, without significant differences between the CKD and ESKD groups. In contrast, the SCD18 index exhibited a progressive decline across the groups, being lowest in the ESKD group.

Regarding elongase indices, the ELOVL5 index was significantly higher in both the CKD and ESKD groups than in controls. The ELOVL6 index demonstrated a progressive increase with advancing kidney disease, being significantly higher in the CKD group than in controls and further elevated in the ESKD group. The Δ5-desaturase index showed no significant differences among the three groups.

### 3.4. Correlation Between Kidney Function and Fatty Acid Composition

**Figure 1:**
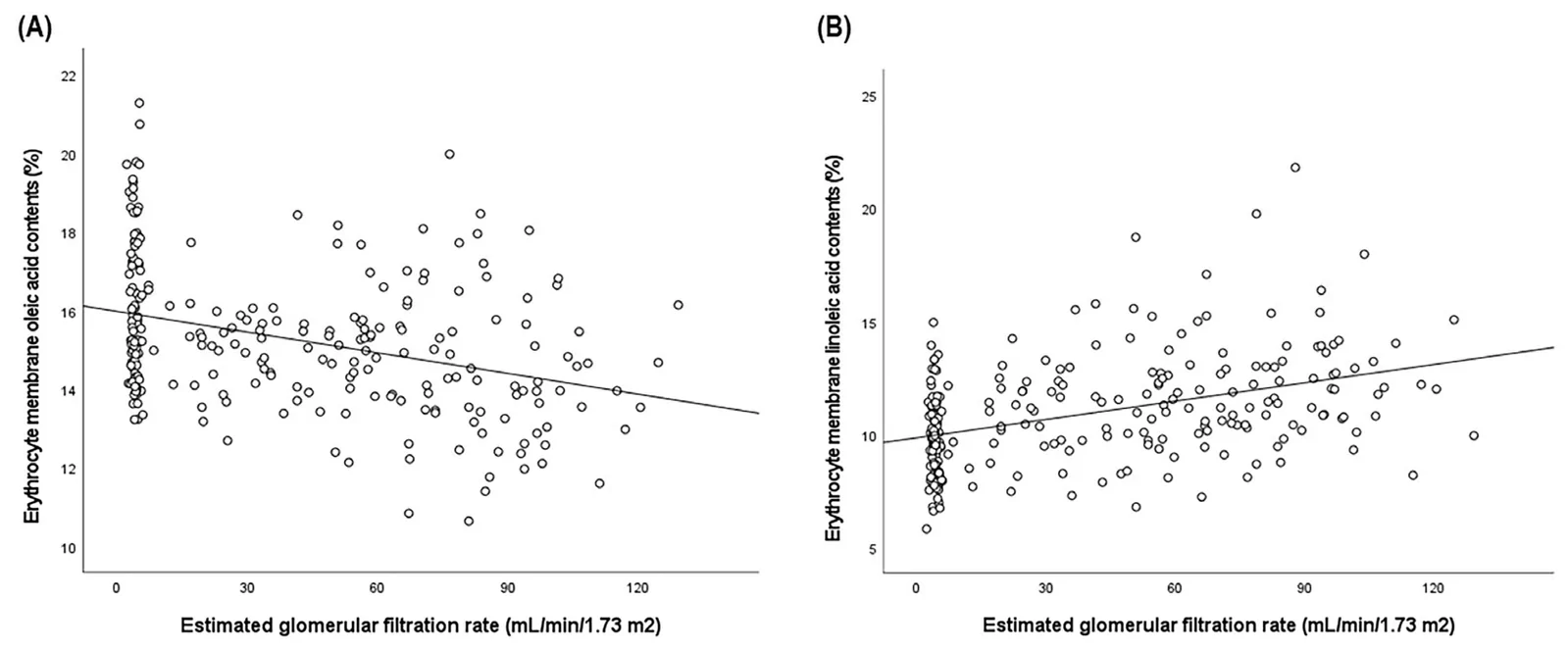
Correlation between estimated glomerular filtration rate and erythrocyte membrane fatty acids; (**A**) oleic acid (*r* = −0.344, *p* < 0.001) and (**B**) linoleic acid (*r* = 0.411, *p* < 0.001).

Correlation analysis revealed significant associations between eGFR and erythrocyte membrane FA composition. Specifically, erythrocyte membrane oleic acid levels were negatively correlated with eGFR (*r* = −0.344, *p* < 0.001), whereas linoleic acid levels showed a positive correlation (*r* = 0.411, *p* < 0.001; Figure 1). Among estimated metabolic indices, eGFR was positively correlated with the desaturase indices SCD16 (*r* = 0.230, *p* < 0.001) and SCD18 (*r* = 0.224, *p* < 0.001), and it was negatively correlated with the elongase index ELOVL6 (*r* = −0.371, *p* < 0.001). No significant correlations were identified between eGFR and the Δ6-desaturase, Δ5-desaturase, or ELOVL5 indices.

### 3.5. Independent Factors Associated with Erythrocyte Membrane Fatty Acid Levels

Multivariable GLMM analysis was conducted to determine the independent association between eGFR and erythrocyte membrane FA levels (Table 3 and Table 4). For erythrocyte membrane oleic acid levels, eGFR remained a significant independent predictor in the multivariable model after adjusting for age, sex, DM, and albumin (β = −0.012, 95% CI: −0.024, −0.0003, *p* = 0.044; Table 3). Similarly, eGFR was independently associated with erythrocyte membrane linoleic acid levels after adjusting for the same clinical confounding variables (β = 0.016, 95% CI: 0.004, 0.028, *p* = 0.012; Table 4). Among the other covariates adjusted in the models, age and female sex showed significant independent associations with linoleic acid levels, whereas DM and serum albumin exhibited no independent associations with either oleic or linoleic acid levels.

In terms of effect magnitude, each 1 mL/min/1.73 m^2^ decline in eGFR was independently associated with a 0.012 percentage-point increase in oleic acid and a 0.016 percentage-point decrease in linoleic acid weight percentage (wt%), corresponding to an estimated absolute shift of 0.72 percentage points and 0.96 percentage points, respectively, across a 60 mL/min/1.73 m^2^ eGFR decline spectrum. Overall, the fixed clinical covariates accounted for approximately 6.7% to 12.9% of the total variance in membrane FAs (Marginal R^2^), while the full models including cohort-level random effects explained 21.4% to 25.0% of the variance (Conditional R^2^). Furthermore, the interaction between eGFR and dialysis status was not statistically significant (*p* = 0.715). In leave-one-cohort-out sensitivity analyses, the effect sizes for the inverse association of eGFR with oleic acid remained generally consistent (β ranging from −0.005 to −0.018), and the positive association with linoleic acid also maintained a consistent direction (β ranging from 0.012 to 0.021), although statistical significance was affected by the exclusion of specific cohorts (Supplementary Table S2). Similarly, in sensitivity analyses specifying study source as a categorical fixed covariate, oleic acid exhibited a borderline inverse association with eGFR (β = −0.009, *p* = 0.072), while linoleic acid remained significantly associated (β = 0.015, *p* = 0.026). When further adjusting for all clinical covariates, these associations did not reach statistical significance, although the directional trajectories were preserved (β = −0.008 for oleic acid and β = 0.005 for linoleic acid). In an additional subgroup analysis limited to non-dialysis participants (Supplementary Table S3), eGFR remained significantly associated with both oleic acid and linoleic acid in univariable models. However, after multivariable adjustment, these associations were no longer statistically significant (*p* = 0.084 for oleic acid and *p* = 0.345 for linoleic acid), although the directional trends were preserved, with age remaining a significant independent factor for linoleic acid levels.

## 4. Discussion

The present study demonstrated that a progressive decline in kidney function is independently associated with alterations in erythrocyte membrane FA composition, characterized by an accumulation of oleic acid and a deficit in linoleic acid. These alterations reflect opposing patterns of estimated desaturase and elongase indices along the continuum of kidney insufficiency. In multivariable GLMM analysis, eGFR remained an independent predictor of both oleic and linoleic acid levels, even after adjusting for age, sex, DM, and serum albumin. These findings indicate that the observed imbalance in FA homeostasis is associated with kidney function impairment beyond secondary clinical comorbidities, suggesting that kidney dysfunction may be linked to systemic lipid metabolic derangements. The observed lipid alterations have substantial clinical implications for the investigated patient population, as altered membrane FA profiles are linked to adverse cardiovascular outcomes and mortality [23]. Given that individual participant data were pooled across eight distinct cohorts, a critical consideration is whether these alterations reflect kidney decline rather than cohort-specific clinical disparities. These findings suggest that declining kidney function may be associated with membrane FA remodeling; however, the attenuation of these associations after multivariable adjustment in the non-dialysis subgroup indicates that other clinical factors, such as age and metabolic status, may also contribute to the observed relationships. In particular, the attenuated multivariable association for linoleic acid in this subgroup was largely attributable to confounding by age, suggesting that age-related nutritional and metabolic factors exert a substantial influence on essential FA levels.

Under physiological conditions, a homeostatic equilibrium between MUFAs and PUFAs is required to maintain cell membrane fluidity and cellular homeostasis [24]. However, the observed shift toward oleic acid-rich and linoleic acid-deficient profiles may compromise membrane fluidity and alter cellular functions [25]. In chronic uremia, such compositional changes are hypothesized to relate to systemic inflammation and oxidative stress, and subsequently accelerate atherogenesis [26]. Although membrane FA alterations relate to cardiovascular risk factors, the lack of cardiovascular endpoints in this study precludes definitive conclusions. These findings serve as a hypothesis-generating observation regarding cardiovascular vulnerability in CKD rather than a mechanistic explanation.

Divergent patterns of the estimated desaturase and elongase indices align with the observed metabolic shifts, including the increase in oleic acid despite reductions in the estimated SCD index. Because the SCD18 index was calculated as a product-to-precursor ratio, this surrogate index is sensitive to substrate availability and does not directly measure enzymatic activity [27]. Under chronic uremic conditions, potential upregulation of ELOVL6 might accelerate the elongation of shorter-chain FAs into longer-chain species, contributing to stearic acid accumulation [28]. This elongation process increases the stearic acid substrate pool, potentially leading to substrate saturation and a consequent reduction in the estimated SCD18 index, even in the presence of elevated oleic acid levels [29]. Beyond substrate dynamics, alternative non-enzymatic mechanisms may contribute to this pattern. Differential membrane turnover rates could favor the preferential incorporation and prolonged retention of oleic acid within erythrocyte membrane phospholipids [30]. Furthermore, altered systemic FA trafficking, such as increased exogenous influx of oleic acid derived from hepatic lipogenesis or peripheral stores, could maintain elevated membrane levels despite suppressed local metabolic indices [31].

These FA alterations, characterized by a metabolic shift toward MUFA synthesis with concomitant depletion of PUFAs, may reflect an underlying metabolically dysregulated state under chronic uremic stress [32]. Elevation of oleic acid has been suggested in cell models as a transient mechanism to mitigate SFA overload by partitioning SFAs into intracellular fractions [33]. However, its progressive accumulation in erythrocyte membranes could serve as a systemic marker of adverse metabolic remodeling rather than as a mediator of protection [34]. Ultimately, the shift toward an oleic acid-rich profile, combined with the concurrent suppression of the Δ6-desaturase index, which is the primary rate limiting pathway in PUFA biosynthesis [35], corresponds to a systemic deficit of long-chain PUFAs. This pathway deficit may shift cell membranes toward a less polyunsaturated profile and is associated with cardiovascular vulnerability in this population [36,37,38].

Specific patterns were also observed in the omega-3 and omega-6 series across clinical stages. While fractions of omega-6 and omega-3 FAs were reduced in the ESKD group, individual FAs showed differential sensitivities to eGFR decline. Linoleic acid and ALA levels decreased progressively from early CKD stages, indicating the early exhaustion of essential precursor FAs [39]. Conversely, EPA and DHA levels were significantly reduced in patients with ESKD [40]. This delayed depletion suggests that peripheral tissues may maintain long-chain omega-3 PUFA levels during early kidney impairment, whereas advanced uremic toxicity at end-stage disease may be associated with accelerated degradation of long-chain species or altered biosynthesis [41]. This terminal depletion is reflected by the lowest omega-3 index and highest AA/EPA ratio in the ESKD population [42]. However, whether dietary restrictions or specific nutritional strategies influence these observational shifts remains speculative and warrants evaluation in prospective trials.

The major strength of the present study is the integration of individual participant data from multiple cohorts using a robust GLMM framework with a standardized gas chromatography protocol. However, this study has several limitations. First, the cross-sectional design of the pooled data precluded establishing causality or bidirectional dynamics between eGFR decline and altered FA profiles. Reverse causation remains a plausible alternative interpretation, as dysregulated FA metabolism may actively promote tubulointerstitial inflammation and fibrosis [43,44]. Prospective longitudinal studies are warranted to clarify these bidirectional dynamics. Second, although multivariable GLMMs were adjusted for key covariates, including age, sex, DM, and serum albumin, residual confounding from unmeasured factors cannot be ruled out, including detailed quantitative dietary habits, nutritional status, use of lipid-lowering therapies, and systemic inflammatory markers. Third, pooling data from eight distinct source cohorts introduced clinical heterogeneity. While random-intercept modeling and sensitivity analyses supported consistent directional trends, potential residual confounding from inter-cohort differences cannot be completely excluded. Additionally, the healthy control group comprised 33 individuals from a single source cohort, whose relatively modest sample size compared with the patient cohorts may limit the generalizability of baseline comparisons. Fourth, patients across the non-dialysis CKD and ESKD spectrum were analyzed as a continuum. Although no significant interaction was observed between eGFR and dialysis status (*p* = 0.715) and separate subgroup analyses were conducted in non-dialysis patients, serum creatinine-based eGFR has inherent limitations as a continuous measure of residual kidney function in maintenance dialysis. Fifth, although erythrocyte membrane FA composition reflects medium-to-long-term FA status, altered erythrocyte survival or turnover dynamics associated with chronic uremia and anemia management could potentially influence membrane lipid incorporation. Finally, desaturase and elongase indices were estimated indirectly using product-to-precursor ratios rather than direct enzymatic assays.

In conclusion, progressive kidney function decline is associated with erythrocyte membrane FA remodeling across the CKD spectrum, predominantly characterized by the accumulation of oleic acid alongside the depletion of essential PUFAs and altered desaturase indices. However, given the attenuation of these associations after multivariable adjustment in the non-dialysis subgroup, these findings should be interpreted with caution. Nevertheless, these observations suggest a potential biological link between kidney impairment and altered systemic lipid metabolism, providing a rationale for future longitudinal and mechanistic studies to evaluate targeted nutritional and metabolic interventions in CKD.

## Figures and Tables

**Table 1:** Clinical blood biochemical analyses of the subjects.

	Controls (*n* = 33)	CKD (*n* = 131)	ESKD (*n* = 118)	*p* Value
Age (years)	48.1 ± 9.2	57.0 ± 14.4 *	56.5 ± 11.8 *	0.001
Male, *n* (%)	19 (57.6)	65 (49.6)	62 (52.5)	0.712
DM, *n* (%)	0	66 (50.4)	64 (54.2)	<0.001
Calcium (mg/dL)	9.1 ± 0.4	9.2 ± 0.5	9.1 ± 0.7	0.148
Phosphorus (mg/dL)	3.5 ± 0.5	3.7 ± 0.6 *	5.3 ± 1.5 *^,^**	<0.001
Hemoglobin (g/dL)	14.2 ± 1.7	12.3 ± 2.0 *	10.9 ± 1.2 *^,^**	<0.001
Glucose (mg/dL)	98.4 ± 12.4	137.5 ± 60.2 *	118.1 ± 80.0 *	0.013
BUN (mg/dL)	15.0 ± 6.9	22.6 ± 10.2 *	69.9 ± 18.4 *^,^**	<0.001
Creatinine (mg/dL)	0.9 ± 0.2	1.5 ± 0.7 *	11.5 ± 3.1 *^,^**	<0.001
eGFR (mL/min/1.73 m^2^)	88.6 ± 12.3	58.5 ± 27.2 *	4.4 ± 1.2 *^,^**	<0.001
Uric acid (mg/dL)	5.6 ± 1.6	6.5 ± 1.8 *	8.1 ± 2.0 *^,^**	<0.001
Albumin (g/dL)	4.5 ± 0.2	3.9 ± 0.7 *	4.0 ± 0.3 *	<0.001
AST (U/L)	N/A	25.3 ± 12.1	19.5 ± 6.4 **	<0.001
ALT (U/L)	N/A	25.3 ± 31.3	17.1 ± 12.7 **	0.021
Total Cholesterol (mg/dL)	191.8 ± 32.3	216.9 ± 74.8 *	158.2 ± 37.7 *^,^**	<0.001
Triglyceride (mg/dL)	167.6 ± 182.0	187.2 ± 113.8	161.9 ± 129.9	0.399
HDL-cholesterol (mg/dL)	52.0 ± 10.4	48.4 ± 14.5	43.7 ± 11.4 *^,^**	0.002
LDL-cholesterol (mg/dL)	105.9 ± 31.8	138.2 ± 41.3 *	90.7 ± 31.5 *^,^**	<0.001
Residual cholesterol (mg/dL)	33.6 ± 23.1	24.1 ± 22.6 *	24.0 ± 21.5 *	0.082
CRP (mg/dL)	N/A	0.3 ± 0.6	0.5 ± 0.8	0.083
Urine protein (g/g)	0.1 ± 0.1	2.6 ± 5.4 *	N/A	0.009
HbA1c (%)	5.6 ± 0.3	7.0 ± 1.2 *	8.7 ± 2.1 *^,^**	<0.001

Data are expressed as means ± SD. * *p* value < 0.05 (mean values are significantly different from controls). ** *p* value < 0.05 (mean values are significantly different from CKD patients). Reference ranges: Calcium, 8.2–10.5 mg/dL; Phosphorus, 2.5–4.5 mg/dL; Hemoglobin, 11–15 g/dL; Glucose, 74–106 mg/dL; BUN, 7–20 mg/dL; Creatinine, 0.51–1.17 mg/dL; eGFR, ≥60 mL/min/1.73 m^2^; Uric acid, 2.4–6.4 mg/dL; Albumin, 3.5–5.2 g/dL; AST, 0–35 U/L; ALT, 0–35 U/L; Total cholesterol, <200 mg/dL; Triglyceride, <150 mg/dL; HDL-cholesterol, ≥40 mg/dL; LDL-cholesterol, <130 mg/dL; CRP, <0.5 mg/dL; Urine protein < 0.15 g/g; HbA1c 3.9–6.1%. DM, diabetes mellitus; BUN, blood urea nitrogen; eGFR, estimated glomerular filtration rate; AST, aspartate aminotransferase; ALT, alanine aminotransaminase, HDL-cholesterol, high-density lipoprotein cholesterol; LDL-cholesterol, low-density lipoprotein cholesterol; CRP, C-reactive protein; HbA1c, Glycosylated hemoglobin; CKD, chronic kidney disease; ESKD, end-stage kidney disease; N/A, not available.

**Table 2:** Comparison of erythrocyte membrane fatty acids content according to kidney function.

	Controls (*n* = 33)	CKD (*n* = 131)	ESKD (*n* = 118)	*p* Value
FA composition (wt%)				
Saturated	39.0 ± 1.0	41.0 ± 0.5	45.1 ± 0.5 *^,^**	<0.001
Myristic	0.60 ± 0.04	0.37 ± 0.02 *	0.37 ± 0.02 *	<0.001
Palmitic	24.5 ± 0.6	23.7 ± 0.3	24.8 ± 0.3 **	0.025
Stearic	13.6 ± 0.5	16.6 ± 0.2 *	19.5 ± 0.3 *^,^**	<0.001
Lignoceric	0.27 ± 0.04	0.38 ± 0.02 *	0.48 ± 0.02 *^,^**	<0.001
Monounsaturated	16.3 ± 0.4	16.1 ± 0.2	17.5 ± 0.2 *^,^**	<0.001
Palmitoleic	1.9 ± 0.1	0.59 ± 0.06 *	0.54 ± 0.07 *	<0.001
Trans-oleic	1.7 ± 0.1	0.42 ± 0.04 *	0.52 ± 0.04 *	<0.001
Oleic	13.9 ± 0.3	15.0 ± 0.2 *	16.0 ± 0.2 *^,^**	<0.001
Polyunsaturated	41.9 ± 1.3	41.9 ± 0.6	36.4 ± 0.7 *^,^**	<0.001
Omega-6	28.4 ± 0.8	29.1 ± 0.4	25.8 ± 0.4 *^,^**	<0.001
Linoleic	13.3 ± 0.4	11.7 ± 0.2 *	9.9 ± 0.2 *^,^**	<0.001
AA	11.5 ± 0.6	13.1 ± 0.3 *	11.6 ± 0.3 **	<0.001
Omega-3	13.5 ± 0.7	12.8 ± 0.3	10.6 ± 0.4 *^,^**	<0.001
Alpha-linolenic	0.37 ± 0.03	0.27 ± 0.02 *	0.21 ± 0.02 *^,^**	<0.001
EPA	2.1 ± 0.2	1.8 ± 0.1	1.4 ± 0.1 *^,^**	<0.001
DHA	8.6 ± 0.4	8.2 ± 0.2	6.6 ± 0.2 *^,^**	<0.001
Omega-3 index	10.7 ± 0.5	10.0 ± 0.2	8.0 ± 0.3 *^,^**	<0.001
AA/EPA	6.0 ± 1.2	10.2 ± 0.5 *	11.1 ± 0.6 *	<0.001
Omega-6/Omega-3	2.2 ± 0.2	2.4 ± 0.1	2.9 ± 0.1 *^,^**	<0.001
Estimated metabolic indices				
SCD16	0.080 ± 0.005	0.025 ± 0.003 *	0.022 ± 0.003 *	<0.001
SCD18	1.05 ± 0.03	0.92 ± 0.01 *	0.84 ± 0.02 *^,^**	<0.001
Δ6-desaturase	0.023 ± 0.002	0.013 ± 0.001 *	0.015 ± 0.001 *	<0.001
Δ5-desaturase	8.9 ± 2.0	11.1 ± 0.9	9.0 ± 1.1	0.294
ELOVL5	8.2 ± 0.9	11.7 ± 0.4 *	11.4 ± 0.5 *	0.003
ELOVL6	0.56 ± 0.02	0.71 ± 0.01 *	0.78 ± 0.01 *^,^**	<0.001

Data are expressed as estimated marginal means ± standard error, adjusted for age, sex, and DM using a mixed-effect model with study source as a random intercept. * *p* value < 0.05 (mean values are significantly different from controls). ** *p* value < 0.05 (mean values are significantly different from CKD patients). AA, arachidonic acid; EPA, eicosapentaenoic acid; DHA, docosahexaenoic acid; DM, diabetes mellitus; SCD, Stearoyl-CoA Desaturase; ELOVL, elongation of very long-chain fatty acids protein.

**Table 3:** Multivariable generalized linear mixed model analysis for factors associated with erythrocyte membrane oleic acid levels.

	Univariable Analysis		Multivariable Analysis	
	β (95% CI)	*p* Value	β (95% CI)	*p* Value
eGFR (mL/min/1.73 m^2^)	−0.011 (−0.030, 0.009)	0.162	−0.012 (−0.024, −0.0003)	0.044
Age (years)	0.004 (−0.014, 0.021)	0.680	−0.005 (−0.026, 0.015)	0.605
Male, *n* (%)	0.191 (−0.375, 0.757)	0.445	−0.288 (−0.698, 0.122)	0.168
DM, *n* (%)	0.474 (−0.258, 1.206)	0.173	−0.224 (−0.673, 0.224)	0.326
Albumin, (g/dL)	−0.277 (−1.061, 0.508)	0.483	−0.340 (−0.728, 0.048)	0.086

β represents the unstandardized regression coefficient, indicating the change in erythrocyte oleic acid weight percentage (wt%) in percentage points per unit change in the independent variable. CI, confidence interval; eGFR, estimated glomerular filtration rate; DM, diabetes mellitus.

**Table 4:** Multivariable generalized linear mixed model analysis for factors associated with erythrocyte membrane linoleic acid levels.

	Univariable Analysis		Multivariable Analysis	
	β (95% CI)	*p* Value	β (95% CI)	*p* Value
eGFR (mL/min/1.73 m^2^)	0.024 (0.011, 0.038)	<0.001	0.016 (0.004, 0.028)	0.012
Age (years)	−0.047 (−0.075, −0.019)	0.001	−0.040 (−0.064, −0.017)	<0.001
Male, *n* (%)	−0.519 (−1.528, 0.491)	0.281	−0.537 (−1.062, −0.013)	0.045
DM, *n* (%)	−0.358 (−1.321, 0.605)	0.394	−0.016 (−0.587, 0.556)	0.957
Albumin (g/dL)	−0.206 (−0.883, 0.471)	0.550	0.078 (−0.526, 0.681)	0.800

β represents the unstandardized regression coefficient, indicating the change in erythrocyte linoleic acid weight percentage (wt%) in percentage points per unit change in the independent variable. CI, confidence interval; eGFR, estimated glomerular filtration rate; DM, diabetes mellitus.

## Data Availability

The data presented in this study are available from the corresponding author upon reasonable request.

## Supplementary Materials

The following supporting information can be downloaded at: https://www.mdpi.com/article/10.3390/membranes16100342/s1, Figure S1: Flowchart of study participant selection and group classification; Table S1: Detailed description of the eight pooled study cohorts; Table S2: Leave-one-cohort-out sensitivity analysis evaluating independent associations of eGFR with erythrocyte oleic acid and linoleic acid levels; Table S3: Univariable and multivariable generalized linear mixed model analyses for factors associated with erythrocyte membrane oleic acid and linoleic acid levels in the non-dialysis population.

## Abbreviations

AA	arachidonic acid
ALA	alpha-linolenic acid
BUN	blood urea nitrogen
CI	confidence interval
CKD	chronic kidney disease
CVD	cardiovascular disease
DHA	docosahexaenoic acid
DM	diabetes mellitus
eGFR	estimated glomerular filtration rate
ELOVL	elongation of very long-chain fatty acids protein
EM means	estimated marginal means
EPA	eicosapentaenoic acid
ESKD	end-stage kidney disease
FA	fatty acid
GLMM	Generalized linear mixed model
HDL	high-density lipoprotein
LDL	low-density lipoprotein
MUFA	monounsaturated FA
PUFA	polyunsaturated FA
SCD	stearoyl-CoA desaturase
SD	standard deviations
SE	standard errors
SFA	saturated fatty acid
Δ5-desaturase	delta-5 desaturase
Δ6-desaturase	delta-6 desaturase
