# Supplementary material for: Declining Kidney Function Is Associated with Altered Erythrocyte Membrane Fatty Acid Profiles and Estimated Enzymatic Indices
Source: Membranes (Basel). 2026 Oct 14;16(10):342. doi: 10.3390/membranes16100342 (PMC13706858; doi:10.3390/membranes16100342)

Supplementary Figure S1. Flowchart of study participant selection and group classification. CKD, chronic kidney disease; ESKD, end-stage kidney disease.

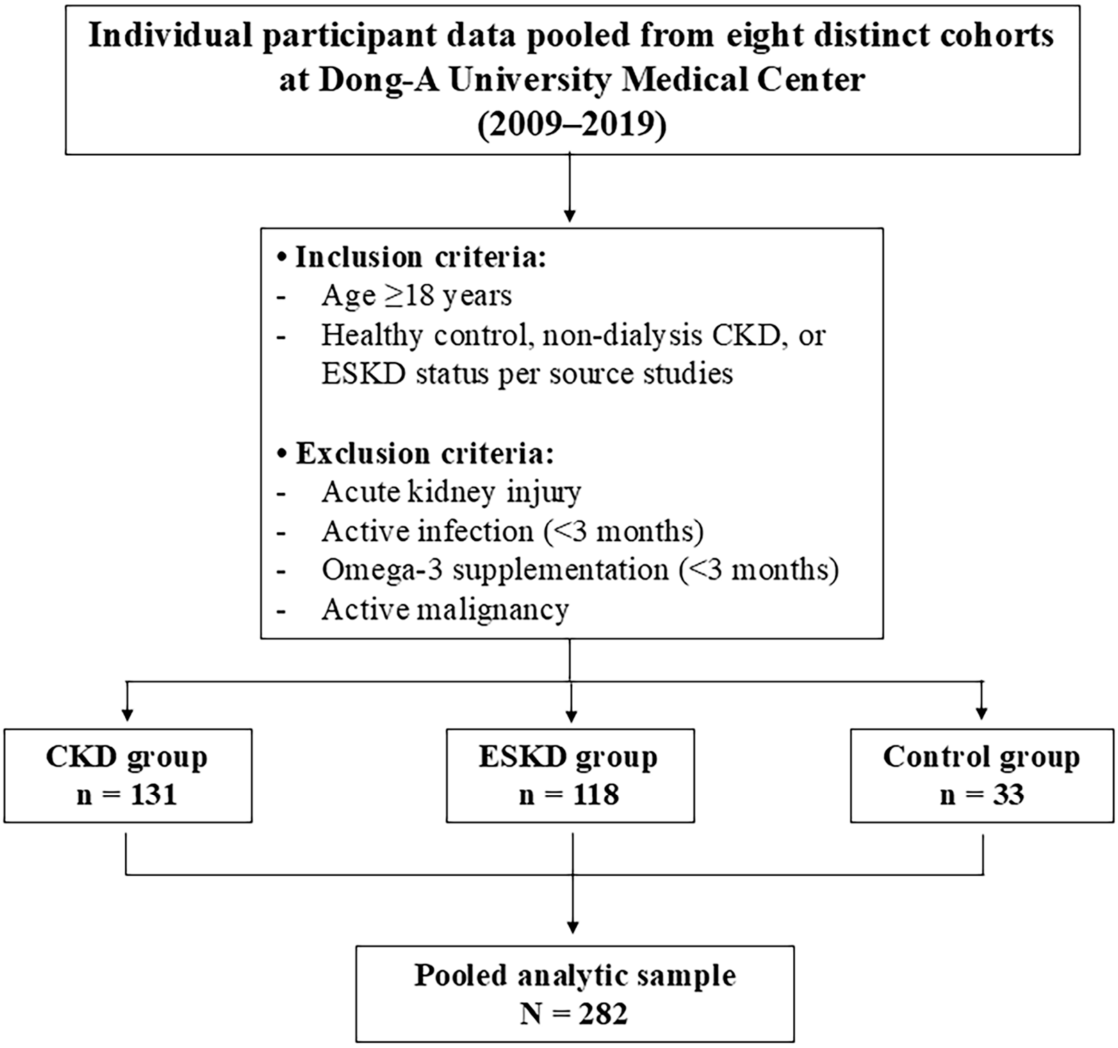

Supplement: Supplementary file 1 [file membranes-16-00342-s001.zip › Supplementary Figure S1.pdf]
