# Supplementary material for: Declining Kidney Function Is Associated with Altered Erythrocyte Membrane Fatty Acid Profiles and Estimated Enzymatic Indices
Source: Membranes (Basel). 2026 Oct 14;16(10):342. doi: 10.3390/membranes16100342 (PMC13706858; doi:10.3390/membranes16100342)
Supplement: Supplementary file 1 [file membranes-16-00342-s001.zip › Supplementary_tables.pdf]

Supplementary Table S1. Detailed description of the eight pooled study cohorts

Source/Citation	Sample size (*n*)	Recruitment period	Eligibility criteria	Clinical group	Dialysis modality
Lee et al. [1]	19	2009-2010	Diabetic nephropathy with proteinuria > 0.3 g/day on ACEi/ARB ≥ 6 months	Non-dialysis CKD	Non-dialysis
Oh et al. [2]	54	2009-2010	Healthy volunteers without kidney disease (n = 33) and maintenance dialysis patients (n = 21)	Healthy control (n=33) & ESKD (n=21)	Non-dialysis /HD&PD
Lee et al. [3]	15	2012	Maintenance HD ≥ 6 months, 25(OH)D <30 ng/mL	ESKD	HD
Lee et al. [4]	55	2013-2014	Maintenance dialysis ≥6 months	ESKD	HD/PD
Lee et al. [5]	45	2017-2019	CKD G1-G5ND, statin-naive	Non-dialysis CKD	Non-dialysis
Kim et al. [6]	28	2015-2016	CKD G1-G5ND, statin-naive	Non-dialysis CKD	Non-dialysis
Hong et al. [7]	27	2015-2016	Maintenance HD ≥ 6 months	ESKD	HD
Unpublished dataset	39	2019	CKD G1-G5ND (IRB No. DAUHIRB-18-237)	Non-dialysis CKD	Non-dialysis

ACEi, angiotensin-converting enzyme inhibitor; ARB, angiotensin II receptor blocker; CKD, chronic kidney disease; ESKD, end-stage kidney disease; G1–G5ND, stages 1–5 non-dialysis; HD, hemodialysis; PD, peritoneal dialysis; 25(OH)D, 25-hydroxyvitamin D. Overlapping dialysis participants across historical study cohorts were systematically identified and excluded prior to statistical pooling.

Supplementary Table S2. Leave-one-cohort-out sensitivity analysis evaluating independent associations of eGFR with erythrocyte oleic acid and linoleic acid levels

Excluded Source[a]	Remaining sample size (*n*)	Oleic acid β[b] (95% CI)	*p* value	Linoleic acid β (95% CI)	*p* value
Full model	282	-0.012 (-0.024, -0.0003)	0.044	0.016 (0.004, 0.028)	0.012
Lee et al. [1]	263	-0.007 (-0.020, 0.007)	0.338	0.019 (0.002, 0.036)	0.034
Oh et al. [2]	228	-0.005 (-0.018, 0.008)	0.465	0.015 (0.001, 0.028)	0.035
Lee et al. [3]	267	-0.009 (-0.022, 0.005)	0.230	0.015 (0.002, 0.028)	0.026
Lee et al. [4]	227	-0.006 (-0.016, 0.004)	0.217	0.012 (-0.002, 0.026)	0.086
Lee et al. [5]	237	-0.013 (-0.027, 0.001)	0.077	0.017 (-0.006, 0.041)	0.128
Kim et al. [6]	254	-0.013 (-0.029, 0.002)	0.096	0.021 (0.007, 0.034)	0.003
Hong et al. [7]	255	-0.018 (-0.029, -0.006)	0.002	0.014 (0.001, 0.027)	0.033
Unpublished dataset	243	-0.013 (-0.028, 0.002)	0.079	0.017 (0.004, 0.030)	0.012

[a]All multivariable linear mixed-effects models were adjusted for age, sex, diabetes mellitus, and serum albumin as fixed effects, with study source incorporated as a random intercept. [b]β represents the unstandardized regression coefficient, indicating the absolute change in erythrocyte fatty acid weight percentage (wt%) per 1 mL/min/1.73 m^2 change in eGFR. *CI,* confidence interval.

Supplementary Table S3. Univariable and multivariable generalized linear mixed model analyses for factors associated with erythrocyte membrane oleic acid and linoleic acid levels in the non-dialysis population.

	Oleic acid		Linoleic acid	
	β (95% CI)	*p* value	β (95% CI)	*p* value
Univariable analysis				
eGFR (mL/min/1.73 m^2)	-0.010 (-0.019, 0.000)	0.044	0.019 (0.005, 0.033)	0.007
Multivariable analysis				
eGFR (mL/min/1.73 m^2)	-0.009 (-0.019, 0.001)	0.084	0.009 (-0.010, 0.027)	0.345
Age (years)	-0.003 (-0.023, 0.018)	0.789	-0.053 (-0.083, -0.022)	<0.001
Male, n (%)	-0.426 (-1.022, 0.619)	0.160	0.304 (-0.420, 1.029)	0.408
DM, n (%)	-0.217 (-0.811, 0.376)	0.471	-0.262 (-1.713, 1.189)	0.722
Albumin (g/dL)	-0.417 (-0.818, -0.016)	0.042	-0.003 (-0.689, 0.684)	0.994

β represents the unstandardized regression coefficient, indicating the change in erythrocyte linoleic acid weight percentage (wt%) in percentage points per unit change in the independent variable. *CI,* confidence interval; *eGFR*, estimated glomerular filtration rate; *DM*, diabetes mellitus.

References

1. Lee, S.M.; Chung, S.H.; Park, Y.; Park, M.K.; Son, Y.K.; Kim, S.E.; An, W.S. Effect of Omega-3 Fatty Acid on the Fatty Acid Content of the Erythrocyte Membrane and Proteinuria in Patients with Diabetic Nephropathy. *Int J Endocrinol* **2015**, *2015*, 208121.
2. Lee, S.M.; Son, Y.K.; Kim, S.E.; An, W.S. The effects of omega-3 fatty acid on vitamin D activation in hemodialysis patients: a pilot study. *Mar Drugs* **2015**, *13*, 741-755.
3. Lee, S.M.; Son, Y.K.; Kim, S.E.; Kim, Y.H.; Park, Y.; An, W.S. Effect of pravastatin on erythrocyte membrane fatty acid contents in patients with chronic kidney disease. *Kidney Res Clin Pract* **2021**, *40*, 392-400.
4. Kim, M.; Kim, S.E.; Lee, S.M.; An, W.S. Effect of pitavastatin on erythrocyte membrane fatty acid content in patients with chronic kidney disease: two-arm parallel randomized controlled trial. *J Yeungnam Med Sci* **2024**, *41*, 188-195.
5. Lee, S.M.; Lee, H.W.; Son, Y.K.; Kim, S.E.; An, W.S. Abdominal aortic calcification score among several vascular calcification scores of plain radiograph is the most reliable predictor of severe coronary artery calcification in dialysis patients. *Ren Fail* **2017**, *39*, 729-735.
6. Oh, J.S.; Kim, S.M.; Sin, Y.H.; Kim, J.K.; Park, Y.; Bae, H.R.; Son, Y.K.; Nam, H.K.; Kang, H.J.; An, W.S. Comparison of erythrocyte membrane fatty acid contents in renal transplant recipients and dialysis patients. *Transplant Proc* **2012**, *44*, 2932-2935.
7. Hong, J.M.; Kim, J.K.; Yang, D.E., Park, Y.; Lee, S.M.; An, W.S. Effect of stir-fried versus non-stir-fried olive oil in cooked food on erythrocyte membrane fatty acid composition in hemodialysis patients: a randomized controlled trial. *Kosin Med J* **2026**, 231-239.
